# Emerging Cellular Therapies: T Cells and Beyond

**DOI:** 10.3390/cells8030284

**Published:** 2019-03-26

**Authors:** Stephen Todryk, Agnieszka Jozwik, Julian de Havilland, Joanna Hester

**Affiliations:** 1 Department of Applied Sciences, Faculty of Health & Life Sciences, Northumbria University, Newcastle upon Tyne NE1 8ST, UK; 2 Institute of Cellular Medicine, Newcastle University, Newcastle upon Tyne NE2 4HH, UK; 3 Department of Haematological Medicine, King’s College London, London SE5 9NU, UK; ajozwik@kcl.ac.uk; 4 Newcastle Biomedicine Cellular Therapies Facility, International Centre for Life, Newcastle University, Newcastle upon Tyne NE1 3BZ, UK; julian.de-havilland@ncl.ac.uk; 5 Transplantation Research and Immunology Group, Nuffield Department of Surgical Sciences, University of Oxford, Oxford OX3 9DU, UK; joanna.hester@nds.ox.ac.uk

**Keywords:** T cells, regulatory T cells, cytotoxic T lymphocytes, CARs, adoptive therapy

## Abstract

Cellular therapies, including those based on T cells, are becoming approved options for clinicians treating a range of diseases. Cytotoxic T lymphocytes (CTLs) can be modified ex vivo to express receptors such as chimeric antigen receptors (CARs) or T cell receptors, allowing them to target tumour cells when infused back into patients with particular cancers. CTLs specific for viruses can be purified ex vivo and reinfused into patients transplanted with haematopoietic stem cells to help combat viral reactivation. Regulatory T cells (Tregs) can be expanded ex vivo for infusion into patients with autoimmunity or allergy, or into those at risk of rejecting transplanted cells or tissues, or suffering graft versus host disease. Effector and regulatory T cells can also be generated by infusion of patient-derived dendritic cells (DCs) conditioned in ways to elicit anti-tumour immunity (CTLs) or Tregs. All such therapies are resource-heavy (particularly in process regulation) and so must be initially targeted to patients that have limited treatment options, but also where they have a chance of being effective.

## 1. Introduction

Cellular therapies are fast becoming a viable option for the treatment of numerous diseases, thanks to the progress of technologies that enhance capabilities and reduce costs. They are typically used only when standard treatments have failed, since they are intensive in terms of skilled labour, reagents and facilities; are usually tailored to the patient; and thus remain expensive and limited in availability compared to off-the-shelf medications. Therapies that have their basis in immune cells benefit from the potency of such cells, together with a mechanistic understanding of their often complex action. Immunologists have always recognized the key role of T cells in health and disease. Indeed, the importance of T cells clinically is highlighted when they are absent or dysfunctional, such as in certain primary (genetic) or secondary (induced) immunodeficiencies, where viral infections are prevalent [1]. Less obvious is their beneficial role as effector T cells in anti-cancer immunity [2], and the role of regulatory T cells (Tregs) in ameliorating inflammatory immune responses when not needed, such as in autoimmunity, transplantation [3], graft versus host disease (GvHD) [4] and allergy [5]. T cell therapies under current use or investigation involve manipulation of T cells in all these contexts, mainly ex vivo followed by reinfusion, using a range of highly regulated technological approaches, and are at various stages of clinical development. This Special Issue of *Cells*, entitled Emerging Cellular Therapies: T Cells and Beyond, aims to highlight these approaches and applications, touch upon their technical and mechanistic bases, and give an update on their current status and efficacy. Future paths that such cellular therapies might take will also be discussed. This editorial signposts readers to the key areas of cellular therapies relating mainly to T cells, but does not extensively review them.

## 2. Anti-Cancer Therapy

T cell-based cancer therapies have had a recent resurgence through the approval and use of immune checkpoint inhibitors [6]. These inhibitors, mostly monoclonal antibodies, allow tumour-specific T cells to react against immunogenic/antigenic tumours (such as melanoma) that are usually inhibited by PD-1 and CTLA-4 checkpoints; these receptors usually being engaged with ligands on target tumour cells or other associated cells. However, immune cell-based cellular therapies go back a long way for cancer. Patient blood has been used to extract, expand and/or differentiate non-specific lymphokine-activated killer (LAK) cells, and tumour-specific T cells, with demonstrated efficacy against certain tumours when reinfused [7]. Resected tumours themselves have been used to extract tumour-infiltrating lymphocytes (TILs) which, when further activated and reinfused, have also demonstrated efficacy [8]. In some instances, IL-2 has been administered alongside these therapies. However, issues with toxicity, including off-target cytotoxicity, have hampered these approaches. For some time, cancer vaccines have been available, made up of patient-derived tumour cells transfected to secrete cytokines (e.g., Granulocyte-Macrophage Colony-Stimulating Factor) which make the cancer antigens more immunogenic [9]. These cells are irradiated and returned to the patient, and have demonstrated some efficacy in certain patients with immunogenic and culturable tumours. Patient-derived dendritic cells differentiated in vitro and pulsed with autologous tumour antigens (lysates) or with proteins and peptides reproducing tumour antigens, have generated tumour-specific T cells when reinfused into patients, which have afforded responses. Limitations are due to the difficulty in generating anti-tumour immunity and to the checkpoints in place to avoid autoimmunity. A logical step could be to combine therapies such as cancer vaccines with checkpoint inhibitors. 

Another approach that has recently received approvals from the Food and Drug Administration (FDA) and the European Medicines Agency (EMA) is the use of chimeric antigen receptor (CAR)-T cells, with two products on the market—axicabtagene ciloleucel (Yescarta) and tisagenlecleucel (Kymriah). These T cells, derived either from patient (autologous) or from healthy donor (allogeneic), are modified by introduction (by lentiviral or retroviral systems, or by transposons) of anti-cancer receptor genes, a single chain variable scFV fragment, and additionally carrying intracellular signalling domain CD3ζ and costimulatory domains 41BB or CD28 [10]. Anti-CD19 CAR-T cells were highly successful in the treatment of B-cell malignancies, especially acute lymphoblastic leukaemia (up to 93% response), but with much lower response rates in lymphomas. These data show that CAR-T cell treatment outside of B-ALL can bring further challenges, especially in the case of solid tumours where antigen expression is poor and highly heterogeneous, and cancer microenvironment constitutes physical barriers as well as immunosuppressive environment impairing CAR-T cell performance. Despite these challenges, the CAR-T cell field is developing rapidly with new technologies aimed at upscaling the production processes and making CAR-T cells an off-the-shelf product using the allogeneic approach (13). Initial success brings hope to those cancer patients who have exhausted all other therapeutic options, and the field is making further improvements to CAR-T cell design to make these therapies more consistent, curative and applicable to solid tumours.

## 3. Anti-Viral T Cells

Transplantation of organs, tissues or cells presents the recipient with several issues that clinicians need to manage. A common factor is the immune suppression given to avoid T cell-mediated rejection of the foreign cells. Recipients are invariably at risk of viral infection or reactivation. In the case of Haematopoietic Stem Cell Therapy for cancer, the recipient’s immune system is ablated with drugs pre-transplant since the malignancies it is treating are usually immune cell-derived. Reconstitution of the recipient with donor T cells gives rise to unwanted GvHD together with the desired graft versus leukaemia effect. These outcomes must be carefully managed with immune suppression. Pre-emptive antiviral drugs are also given but patients suffer from toxicity, and a proportion of viral infections are refractory to drug treatments. The application of virus-specific T cells generated from the donor allows a more targeted approach that gives rise to 70% efficacy in recipients with minimal side effects [11]. An issue is the generation of sufficient cells of the correct quality. This is being addressed by the possibility of using banks of stored T cells that are specific for viral antigenic peptides restricted to a single Human Leukocyte Antigen that matches the recipient. Furthermore, T-cell products containing T cells of multiple specificities against the main viral threats are being developed. The commonly problematic virus in transplantation is Cytomegalovirus, whereas Epstein-Barr Virus, Adenovirus, Human Herpes Virus 6, BK virus and JC virus are also threats. 

## 4. Regulatory T Cells (Tregs)

Tregs, typically defined by high levels of surface IL-2R**α** (CD25) and intracellular transcription factor FOXP3, are vital to down-regulating unwanted harmful immunity, often via CTLA-4 and TGF**β**. Autoimmune diseases are characterized by dysregulated immune response towards self-antigens, whereas in organ transplantation the host’s immune system can recognize and attack the transplanted allogeneic organ. In both indications, current standard treatment involves prolonged, often life-long, treatment with immunosuppressive drugs. Regulatory T cells have shown much promise in their ability to inhibit immune responses in vitro and in the animal models and can be utilized to reduce the need for pharmacological immunosuppression. Recent advances in the understanding of Treg biology, isolation and expansion of human regulatory T cells, and in the development of Good Manufacturing Process (GMP)-compliant cell production methods, have led to a series of phase I clinical trials using Tregs in autoimmunity and transplantation (reviewed by Bluestone and Tang [12] and Kawai et al. [13], respectively). The majority of currently used approaches utilize patients’ own CD25^+^CD4^+^ T cells expanded polyclonally with TCR and CD28 stimulation, in the presence of IL-2 and rapamycin. Challenges and hurdles in Treg cellular therapy include a lack of antigen-specificity of the cell product (diluting its effect), cell survival and stability in vivo, as well as production costs. Newer approaches include the development of antigen-specific Tregs, including CAR-Tregs, improvements in the initial purity and phenotypic stability, and genetic modifications to increase cell potency (e.g., upregulating IL-2R and FOXP3) [14]. Most of these are still at the pre-clinical stages [15]. The next few years will provide clinical data on the efficacy of Treg therapy in multiple indications, and improvements in cell product and clinical protocols.

## 5. Regulatory Issues

Being classed as Advanced Therapy Medicinal Products by the EMA, T cells for adoptive therapy present a range of regulatory hurdles that require negotiation [16]. These regulations contribute significantly to the cost and availability of the treatment and the time taken to generate the products. The high-tech production processes need to be pre-defined to conform to GMP and must be overseen by qualified individuals. The manufacture must take place in a certified controlled environment governed by strict procedures that prevent contamination of the product with biological or chemical hazards. Quality control must be carried out on the product before release to clinicians, ensuring purity and potency of the product. The presence of viruses (CMV, EBV, HIV, etc.) within the T cell product must be considered. Typically, patients who are positive for CMV or EBV have been allowed to receive HSCT from equally positive donors, precluding the chance of contracting the virus. A similar process could apply for adoptive isolated T cells. The potency of the T cell product may not only be dictated by the number of cells present but also by the quality of the T cells of which it is comprised. The latter requires the biology of the T cells to be better understood in these contexts. 

## 6. Conclusions

The power of T cells is starting to be harnessed in T cell adoptive therapies. Such therapies, that are often personalized, require high-tech production that is resource-heavy. Thus, they are currently reserved for situations where other, usually standard, treatments have failed. It may be more challenging to demonstrate efficacy in such patients, but accumulating data will likely identify situations where these Regenerative Medicine treatments can become mainstream.
